# Catatonic syndrome: origin, diagnosis, treatment and iatrogenesis. Case report

**DOI:** 10.1192/j.eurpsy.2025.1888

**Published:** 2025-08-26

**Authors:** M. Viana Perez, Á. De Vicente Blanco, A. Muñoz San Jose, Á. Esquembre García, C. Herranz Serfaty, M. E. Sanchez-Escalonilla Relea, M. Velasco Santos

**Affiliations:** 1 Psychiatry; 2 Hospital Universitario La Paz, Madrid, Spain

## Abstract

**Introduction:**

The catatonic syndrome is a heterogeneous syndrome that manifests with a variety of symptoms, whose management is not clearly predefined despite being a clinically diagnosable entity. It is a frequently underdiagnosed and undertreated condition that can lead to the death of the patient, and which originates from a large number of psychiatric and organic pathologies.

**Objectives:**

To present a case highlighting the most significant and representative findings typically observed in catatonic syndrome, as well as to highlight the most relevant data regarding the origin, diagnosis and treatment of this entity.

**Methods:**

This case report describes a single patient. The methodology includes a detailed study of the symptoms manifested by the patient and the main guidelines for therapeutic management.

**Results:**

In this poster, the case of a 24-year-old man who comes to the emergency room with what appears to be catatonic syndrome is presented. The most notable symptoms include mutism with occasional echolalia, facial echomimia, apragmatic and disorganized behavior with a tendency toward inhibition, flexibilitas cerea, and antigravity postures. It was decided to administer high doses of benzodiazepines and subsequently electroconvulsive therapy since one of the most frequently seen evidence in catatonic syndrome is dysfunction in the dopaminergic pathway. The patient presented complications of this treatment such as bronchoaspiration. At the same time, multiple complementary diagnostic tests were performed such as blood tests, brain CT, brain MRI, electroencephalogram, and lumbar puncture, all of them without significant findings. Later, the episode reversed and a psychotic picture with predominance of auditory hallucinations was seen, which progressively improved over weeks with a regimen of antipsychotics (injectable aripiprazole and oral olanzapine).

**Conclusions:**

It is therefore concluded that it would be beneficial for it to be more widely represented in treatment guidelines and clinical trials, which would lead to easier and faster clinical decision-making. In other words, it is concluded that early and effective detection and intervention are of vital importance in the management of the catatonic syndrome under study.

**Disclosure of Interest:**

None Declared

